# Enhanced prefrontal nicotinic signaling as evidence of active compensation in Alzheimer’s disease models

**DOI:** 10.1186/s40035-024-00452-7

**Published:** 2024-12-03

**Authors:** Saige K. Power, Sridevi Venkatesan, Sarah Qu, JoAnne McLaurin, Evelyn K. Lambe

**Affiliations:** 1 Department of Physiology, Temerty Faculty of Medicine, University of Toronto, Toronto, ON M5S 1A8 Canada; 2 Department of Laboratory Medicine and Pathobiology, Temerty Faculty of Medicine, University of Toronto, Toronto, ON M5S 1A8 Canada; 3 Biological Sciences, Hurvitz Brain Sciences Research Program, Sunnybrook Research Institute, Toronto, ON M4N 3M5 Canada; 4 Department of Obstetrics and Gynaecology, Temerty Faculty of Medicine, University of Toronto, Toronto, ON M5G 1E2 Canada; 5 Department of Psychiatry, Temerty Faculty of Medicine, University of Toronto, Toronto, ON M5T 1R8 Canada

**Keywords:** Alzheimer’s disease, Cognitive reserve, Prefrontal cortex, Acetylcholine, Nicotinic receptors, Optogenetics, Electrophysiology, Acetylcholinesterase inhibitor, Compensation

## Abstract

**Background:**

Cognitive reserve allows for resilience to neuropathology, potentially through active compensation. Here, we examine ex vivo electrophysiological evidence for active compensation in Alzheimer’s disease (AD) focusing on the cholinergic innervation of layer 6 in prefrontal cortex. Cholinergic pathways are vulnerable to neuropathology in AD and its preclinical models, and their modulation of deep layer prefrontal cortex is essential for attention and executive function.

**Methods:**

We functionally interrogated cholinergic modulation of prefrontal layer 6 pyramidal neurons in two preclinical models: a compound transgenic AD mouse model that permits optogenetically-triggered release of endogenous acetylcholine and a transgenic AD rat model that closely recapitulates the human trajectory of AD. We then tested the impact of therapeutic interventions to further amplify the compensated responses and preserve the typical kinetic profile of cholinergic signaling.

**Results:**

In two AD models, we found potentially compensatory upregulation of functional cholinergic responses above non-transgenic controls after onset of pathology. To identify the locus of this enhanced cholinergic signal, we dissected key pre- and post-synaptic components with pharmacological strategies. We identified a significant and selective increase in post-synaptic nicotinic receptor signalling on prefrontal cortical neurons. To probe the additional impact of therapeutic intervention on the adapted circuit, we tested cholinergic and nicotinic-selective pro-cognitive treatments. Inhibition of acetylcholinesterase further enhanced endogenous cholinergic responses but greatly distorted their kinetics. Positive allosteric modulation of nicotinic receptors, by contrast, enhanced endogenous cholinergic responses and retained their rapid kinetics.

**Conclusions:**

We demonstrate that functional nicotinic upregulation occurs within the prefrontal cortex in two AD models. Promisingly, this nicotinic signal can be further enhanced while preserving its rapid kinetic signature. Taken together, our work suggests that compensatory mechanisms are active within the prefrontal cortex that can be harnessed by nicotinic receptor positive allosteric modulation, highlighting a new direction for cognitive treatment in AD neuropathology.

**Supplementary Information:**

The online version contains supplementary material available at 10.1186/s40035-024-00452-7.

## Introduction

Cognitive reserve denotes an individual’s ability to withstand a higher degree of neurological decline before exhibiting cognitive impairment [1]. Lifestyle factors have been shown to promote cognitive reserve, decreasing risk of dementia and slowing cognitive decline in Alzheimer’s disease (AD) [2–4]. The impact of these factors is not limited to disease onset but persists even as neuropathology worsens [5–9]. In fact, cognitive reserve is hypothesized to allow for compensation during neuropathology [10, 11], generating a new or stronger response to sustain network recruitment [12, 13]. In AD and its preclinical models, there are now several diverse examples of neuronal compensation leading to cognitive maintenance [14, 15], but compensatory capacity in the cholinergic system has not been systematically evaluated.

Cholinergic pathways in prefrontal cortex are well positioned to contribute to mechanisms of cognitive reserve in AD [16, 17]. This modulation of layer 6 of prefrontal cortex is essential for attention, executive function, and learning [18–24]. Pro-cholinergic treatments improve cognition preclinically [25–27] and slow cognitive decline clinically [28–31]. While the remodelling of the cholinergic system in AD has been extensively probed, key clinical [32–34] and preclinical studies [35–37] focused on cholinergic neurodegeneration and synaptic loss. There has been relatively little functional investigation in early to mid-disease synaptic changes, except in the most swiftly-progressing genetic models of AD that preclude the use of littermate controls [38–47]. To detect and systematically investigate the mechanisms of compensatory plasticity, it is essential to use a well-charted model with appropriately-matched non-transgenic controls.

In this study, we used optogenetics and whole-cell electrophysiology ex vivo to investigate prefrontal acetylcholine pathways innervating layer 6 pyramidal neurons. In models of AD from two different species, we found a strong and significant upregulation of these cholinergic responses above non-transgenic levels after onset of pathology. To probe the mechanisms underlying this functional upregulation, we systematically manipulated pre- and postsynaptic elements of the cholinergic synapse. We discovered the upregulation is specific to nicotinic receptor signalling. Lastly, we assessed the functional impact of therapeutic interventions on cholinergic responses, contrasting current standard AD treatment with a novel nicotinic treatment. Both manipulations improved endogenous cholinergic responses, but only the targeted nicotinic treatment preserved the typical rapid timing essential for cue detection. Overall, we demonstrate evidence for active cholinergic upregulation and put forward a mechanism of cognitive compensation that remains accessible to further potentiation with treatment.

## Materials and methods

### Animals

Animal experiments were approved by the University of Toronto Temerty Faculty of Medicine Animal Care Committee and conducted in accordance with the guidelines of the Canadian Council on Animal Care (protocols #20011621, #20011658, #20011796). TgCRND8 transgenic mice expressing double human amyloid precursor protein (*APP*) Swedish and Indiana mutations (KM670/671NL/V717F) under the Prp gene promoter [48] on a C57BL/6 background were crossed with transgenic mice expressing channelrhodopsin (ChR2) in cholinergic afferents (ChATChR2; RRID:IMSR_JAX:014546, Jackson Laboratory, Bar Harbor, ME [49]). Cholinergic neurons originating from the nucleus basalis project throughout the brain and densely innervate the deep layers of the prefrontal cortex [49, 50], where we optogenetically stimulated their afferents. This transgenic model expresses ChR2 under the choline acetyltransferase promoter, localizing ChR2 expression to neurons that are cholinergic at the time of recording. This approach avoids expression of ChR2 in neurons that are only transiently cholinergic during development [51]. TgCRND8/ChATChR2 mice were compared to ChATChR2 littermates as controls. Male and female mice were weaned at postnatal day (P)21, separated by sex, and group-housed (2–4 mice per cage) in plastic cages with corn cob bedding, houses for environmental enrichment, and ad libitum access to food and water on a 12-h light/dark cycle with lights on at 7:00 AM. Mice were used at two age groups based on previously described progression [52–56]: an early to mid-disease group and controls, 3–6 months old (*n* = 30, mean 4.7 ± 0.2 months), and a later disease group and controls, 7–12 months (*n* = 26, mean 9.9 ± 0.3 months). Groups were balanced for sex and genotype.

TgF344 AD rats expressing the human *APP* Swedish mutation (KM670/671NL) and presenilin 1 with exon 9 excised, under the mouse prion promoter [57], were outbred on a Fischer strain. TgF344 AD rats were compared to F344 littermates as controls. Rats were bred at Sunnybrook Research Institute (protocol #22655), and housed in plastic cages with corn cobb bedding, 2 animals per cage, with houses and toys for environmental enrichment, on a 12-h light/dark cycle with ad libitum access to food and water. Rats were transferred to the University of Toronto Division of Comparative Medicine and housed for a minimum of 6 weeks before experiments. Rats were used at three age groups based on stages described previously [58–61]: an age group of 8 months for consideration of early AD and controls (*n* = 21, mean 8.4 ± 0.2 months), an age group of 12 months for mid-AD and controls (*n* = 23, mean 12.4 ± 0.2 months), and an age group of 18 months for later-AD and controls (*n* = 26, mean 17.6 ± 0.2 months). Groups were balanced for sex and genotype.

### Electrophysiology and optogenetics

 Animals were euthanized by decapitation following injection of chloral hydrate to render unconscious. This approach was justified because the cholinergic receptor targets of this investigation are sensitive to other anaesthetics [62–67]. The protocols were approved by the Animal Ethics Committee of the Temerty Faculty of Medicine at the University of Toronto (mice: #20011621, #20011658, rats: #20011796). The brain was rapidly removed in 4 °C sucrose ACSF (254 mM sucrose, 10 mM *D*-glucose, 26 mM NaHCO_3_, 2 mM CaCl_2_, 2 mM MgSO_4_, 3 mM KCl, and 1.25 mM Na_2_PO_4_). The 400-μm thick cortical slices of the prefrontal cortex (bregma 2.2–1.1) were obtained on a Dosaka linear slicer (SciMedia, Costa Mesa, CA). Slices were transferred to a prechamber (AutoMate Scientific, Berkeley, CA) where they recovered for at least 2 h in oxygenated (95% O_2_, 5% CO_2_) ACSF (128 mM NaCl, 10 mM *D*-glucose, 26 mM NaHCO_3_, 2 mM CaCl_2_, 2 mM MgSO_4_, 3 mM KCl, and 1.25 mM Na_2_PO_4_) at 30 °C before being used for electrophysiology.

For whole-cell patch-clamp electrophysiology, brain slices were transferred to a chamber mounted on the stage of a BX51WI (Olympus, Richmond Hill, ON, Canada) microscope and perfused with 30 °C oxygenated ACSF at 3–4 ml/min. Layer 6 pyramidal neurons in the prelimbic and anterior cingulate regions of the medial prefrontal cortex were identified by size, morphology, and proximity to white matter, visualized using infrared differential interference contrast microscopy. Recording electrodes were filled with patch solution containing 120 mM K-gluconate, 5 mM MgCl_2_, 4 mM K-ATP, 0.4 mM Na_2_-GTP, 10 mM Na_2_-phosphocreatine, and 10 mM HEPES buffer adjusted to pH 7.33 with KOH. Data were acquired and low-pass filtered at 20 kHz with Axopatch 200b amplifier (Molecular Devices, San Jose, CA) and Digidata 1440 digitizer and pClamp10.3 acquisition software (Molecular Devices). Responses from a homogenous population of layer 6 regular spiking pyramidal neurons [68, 69] were examined in voltage clamp at − 75 mV and in current clamp.

To excite ChR2-containing cholinergic afferents optogenetically (opto-ACh), blue light (470 nm, 2 mW) was delivered in 5-ms pulses with a collimated LED (Thorlabs, Newton, NJ). This opto-ACh stimulus was delivered in a frequency-accommodating (50–10 Hz) eight-pulse train to stimulate cholinergic axons [68, 69] to mimic the activation pattern of cholinergic neurons [70–72]. This opto-ACh protocol elicits direct cholinergic inward current responses resilient to combined application of glutamatergic synaptic blockers [50, 68, 69, 73] and sensitive to combined cholinergic receptor antagonists [68].

### Experimental design, analysis, and statistics

Animals of both sexes were used in pharmacological interventions. All experiments were performed on multiple rodents per intervention, and recordings were made from 1 to 3 neurons per brain slice of prefrontal cortex (2–3 slices from each mouse, and 3–4 slices from each rat). Pharmacological agents were pre-applied for 10 min and co-applied during optogenetic stimuli or exogenous acetylcholine bath application and recovery period. CNQX (50 µM) and D-APV (50 µM) were applied to block glutamatergic synaptic transmission; bicuculline (3 µM) and CGP55845 (1 µM) were applied to block GABA receptors; atropine (200 nM; Sigma-Aldrich) was applied to block muscarinic receptors; dihydro-β-erythroidine (DHβE; 3 µM) to block β2-containing nicotinic receptors; AF-DX 116 (300 nM) to block M2 muscarinic receptors; galantamine hydrobromide (1 µM) to block acetylcholinesterase, and NS9283 (1 µM) to potentiate nicotinic responses. Compounds were from Tocris Biosciences (Toronto, Canada) unless otherwise specified.

Data analysis was performed in Clampfit 10.3 (Molecular Devices) and Axograph and statistical analysis was performed with Graphpad Prism 10. To measure fast-onset kinetics, raw traces were used for calculating rising slope of current response within 50 ms of opto-ACh onset. To measure response magnitude, downsampled traces were used to fit triple exponentials to opto-ACh responses, from which the peak amplitude (pA) and decay constant (s), where applicable, were measured.

Firing rise was measured by the mean increase in firing frequency for the initial 0.5 s following the onset of the opto-ACh stimulus relative to the mean baseline firing frequency (calculated over 2 s before opto-ACh stimulus). Peak firing frequency was measured as the difference between the mean baseline firing frequency and fastest instantaneous firing frequency following opto-ACh stimulus. Genotype differences in opto-ACh responses were assessed with unpaired two-tailed *t*-tests. Pharmacological manipulations to assess synaptic components of the cholinergic response were calculated with unpaired, two-tailed *t*-tests. The effects of therapeutic interventions were assessed with paired, two-tailed *t*-tests. The impact of sex on cholinergic measures and interactions between sex and genotype were analyzed by two-way ANOVA.

To measure currents to exogenous ACh, 1 mM ACh was bath-applied for 15 s. Genotype differences in acetylcholine current magnitude were measured by peak amplitude. Acetylcholine firing response was measured from stimulus onset to peak firing. Both measures were graphed with a cumulative frequency distribution with 10 pA or 10 s bin width, allowing for direct comparison of each age group’s genotype differences. Genotype differences in these measures were calculated with a nonparametric Kolmogorov Smirnov (KS) test.

Intrinsic neuronal properties were assessed by current-clamp and voltage-clamp recordings at baseline and statistically analysed using unpaired two-tailed *t*-tests. To measure intrinsic neuronal excitability, input–output curves were generated by measuring neuronal firing frequency in response to increasing steps of injected current. These data were statistically analysed by performing a second-order polynomial nonlinear regression of input–output curves and a comparison of fit, analysed with an F test.

## Results

### Increased opto-ACh responses in TgCRND8 AD mice at early to mid-disease stage

**Fig. 1:**
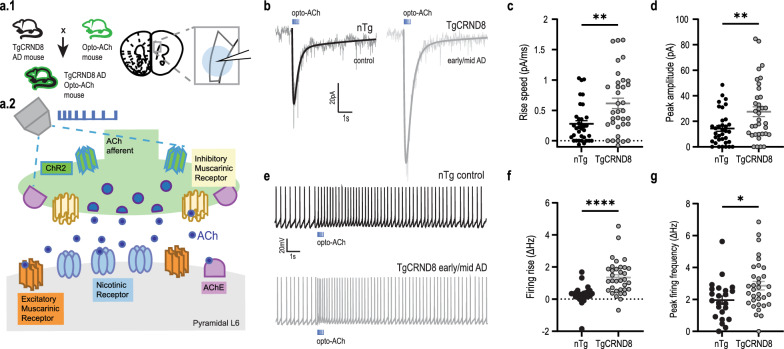
Endogenous opto-ACh responses are upregulated in early to mid-AD. **a.1** Image depicts compound transgenic mouse cross between TgCRND8 AD mouse model and ChAT-ChR2 mouse model expressing channelrhodopsin in cholinergic neurons. Image depicts coronal brain section from these mice, showing the recording electrode positioned over layer 6 of the prefrontal cortex. **a.2** Schematic adapted from Power et al., 2023, represents the cholinergic prefrontal synapse receiving optogenetic stimulus (0.5-s pulse train of decreasing frequency). Afferents release endogenous acetylcholine (ACh) which binds to nicotinic and muscarinic receptors, exciting postsynaptic pyramidal neurons. **b** Representative opto-ACh responses from non-transgenic (nTg) control (black) and TgCRND8 AD (grey) neurons measured in voltage clamp (*V*_m_ =  − 75 mV). **c**, **d** Graphs show significant increases in rise speed (*P* < 0.01) (**c**) and peak amplitude (*P* < 0.01) (**d**) of opto-ACh currents in TgCRND8 early AD responses relative to age-matched controls (3–6 months old). **e** Representative opto-ACh responses from WT control (black) and TgCRND8 AD (grey) neurons measured in current clamp. **f**, **g** Graphs show significant increase in firing rise (*P* < 0.0001) (**f**) and peak firing frequency (*P* < 0.05) (**g**) of opto-ACh firing responses in TgCRND8 early/mid AD responses relative to age-matched controls. Mice aged 3 to 6 months (nTg: 4.5 ± 0.4 months, *n* = 10 animals; 3–4 brain slices per animal, TgCRND8: 4.8 ± 0.3 months,* n* = 10 animals, 3–4 brain slices per animal)

To measure functional changes in the prefrontal cortex cholinergic signals in an AD model, we recorded whole-cell responses to endogenous acetylcholine released optogenetically (opto-ACh; Fig. 1a1, a2) in TgCRND8/ChATChR2 AD mice during early to mid-AD (3 to 6 months) and their ChATChR2 control littermates [52–56]. Cholinergic neurons from the nucleus basalis project throughout the brain and densely innervate the deep layers of the prefrontal cortex [49, 74], where the cholinergic afferents were optogenetically stimulated. Optogenetic responses were recorded from pyramidal neurons in layer 6 of the prelimbic and anterior cingulate areas of the prefrontal cortex. The layer 6 receives dense innervation of cholinergic afferents [74] and shows strong excitatory responses [75]. Responses from a homogenous population of regular spiking pyramidal neurons [68, 69] were examined in voltage-clamp at − 75 mV and in current-clamp. The brief opto-ACh protocol [68, 69, 73] elicited inward current responses resilient to combined application of glutamatergic synaptic blockers CNQX (20 µM) and D-APV (50 µM) in non-transgenic control (opto-ACh; 39.2 ± 14 pA, opto-ACh synaptic blockers: 40.5 ± 14.3 pA; paired *t*-test: *t*_5_ = 0.3, *P* = 0.8) and TgCRND8/ChATChR2 mice (opto-ACh; 82.7 ± 22 pA, opto-ACh synaptic blockers: 80.4 ± 19 pA; paired *t*-test: *t*_4_ = 0.3, *P* = 0.8), and resilient to combined application of GABA receptor blockers bicuculline (3 µM) and CGP (1 µM) in non-transgenic control (opto-ACh; 28.2 ± 6.5 pA, opto-ACh GABA blockers: 24.8 ± 5.9 pA; paired *t*-test: *t*_5_ = 2, *P* = 0.1) and TgCRND8/ChATChR2 mice (opto-ACh; 45.4 ± 11.9 pA, opto-ACh synaptic blockers: 43.8 ± 11.5 pA; paired *t*-test: *t*_7_ = 0.7, *P* = 0.5).

We found a significant increase in the opto-ACh current responses in early to mid-AD mice (3–6 months) (Fig. 1b), reflected by significantly greater rising slope (Fig. 1c, unpaired two-tailed *t-*test: *t* = 3.3, *P* = 0.002, *df* = 68) and peak amplitude (Fig. 1d, unpaired two-tailed *t*-test: *t* = 3, *P* = 0.004, *df* = 68).

This upregulation was also detected in the change of the firing response elicited by opto-ACh (Fig. 1e). Holding the neurons in current clamp and injecting current to elicit ~ 2–3 Hz action potential firing at baseline, opto-ACh elicited a significantly greater increase in the delta initial firing frequency or firing rise (Fig. 1f, unpaired two-tailed *t*-test: *t* = 4.2, *P* < 0.0001, *df* = 54) and the delta peak firing frequency (Fig. 1g, unpaired two-tailed *t*-test: *t* = 2.3, *P* = 0.02, *df* = 54) in AD compared to control.

To delve deeper into the opto-ACh result, we investigated the effect of sex, but did not detect a main effect of this factor (two-way ANOVA, *F*_1, 64_ = 1.4, *P* = 0.2), nor a significant interaction between sex and genotype (*F*_1, 64_ = 1.8, *P* = 0.4) on cholinergic current amplitude.

In addition, there was no difference in intrinsic membrane properties between TgCRND8/ChATChR2 and ChATChR2 mice (Additional file 1: Table S1). We also assessed neuronal excitability by measuring action potential firing rate in response to increasing steps of current injection and generating input–output curves. In the absence of opto-ACh, we found no significant difference in the excitability curves (nonlinear regression, comparison of fit, Additional file 1: Fig. S1a). These findings underscore the relative specificity of opto-ACh in this AD mouse model to increase the excitability of layer 6 pyramidal neurons during early to mid-disease stage.

### Upregulated opto-ACh responses do not persist in later-disease in TgCRND8 AD mouse model

**Fig. 2:**
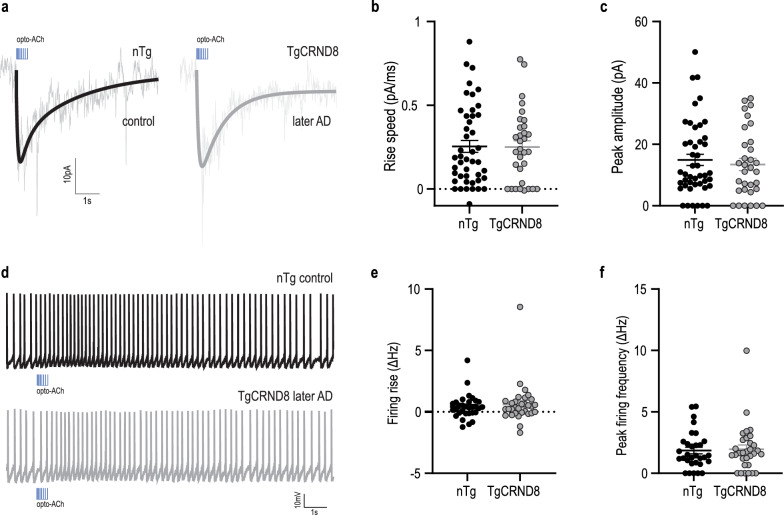
Cholinergic signaling did not differ from non-transgenic in late AD. **a** Representative opto-ACh responses from non-transgenic (nTg) control (black) and TgCRND8 AD (grey) neurons at late stage of disease measured in voltage clamp (*V*_m_ =  − 75 mV). **b**, **c** Graphs show no difference in rising speed (**b**) and peak amplitude (**c**) of opto-ACh currents in TgCRND8 late-AD responses compared to age-matched controls. **d** Representative opto-ACh responses from WT control (black) and TgCRND8 AD (grey) neurons at late stage of disease measured in current clamp. **e**, **f** Graphs show no difference in firing rise (**e**) and peak firing frequency (**f**) of opto-ACh firing responses in TgCRND8 late-AD responses compared to age-matched controls. Mice aged 7–12 months (nTg: 9.3 ± 0.5 months, *n* = 10 animals, 3–4 brain slices per animal, TgCRND8: 10.1 ± 0.6 months, *n* = 10 animals, 3–4 brain slices per animal)

To ask what happens to the AD cholinergic response with progression of neuropathology, we recorded from TgCRND8/ChATChR2 AD mice and their age- and sex-matched non-transgenic control littermates at a more advanced stage of AD. In this older group (7–12 months), the opto-ACh current responses in AD mice were similar to the non-transgenic level (Fig. 2a–c; unpaired two-tailed *t*-test: rising slope *t* = 0.07, *P* = 0.9, df = 75; peak amplitude *t* = 0.5, *P* = 0.6, df = 75). Similar results were found for opto-ACh firing responses (Fig. 2d–f; unpaired two-tailed *t*-test: firing rise *t* = 0.6, *P* = 0.6, df = 60; peak firing *t* = 0.2, *P* = 0.8, df = 60). Cholinergic responses in the model were no longer significantly greater than controls at the later time point.

Consistent with the younger age group, we found no significant effect of sex (two-way ANOVA, *F*_1, 73_ = 0.1, *P* = 0.7) or significant interactions between sex and genotype (*F*_1, 73_ = 2.2, *P* = 0.1) on cholinergic current amplitude.

Further investigation of the intrinsic membrane properties of these neurons showed no significant genotype differences (Additional file 1: Table S1). Regarding neuronal excitability, input–output analysis revealed no significant genotype difference in excitability curves (Additional file 1: Fig. S1b; nonlinear regression, comparison of fit). These results suggest that the prefrontal endogenous cholinergic responses are upregulated in AD mice of early/mid-pathology but not of advanced disease stage.

### Increased cholinergic responses at mid-disease in TgF344 AD rat model

**Fig. 3:**
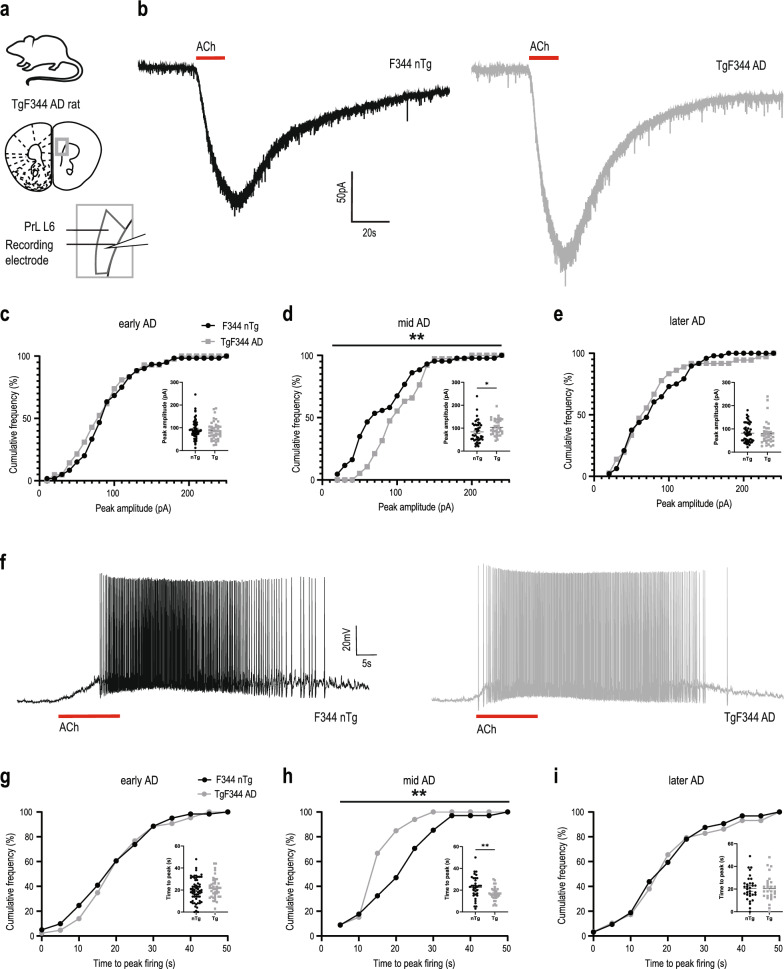
Early cholinergic upregulation conserved in different species and AD models. **a** Image depicts TgF344 AD rat model and a coronal brain section with the recording electrode positioned over layer 6 of the prefrontal cortex. **b** Representative responses to exogenous bath-applied acetylcholine from F344 non-transgenic (nTg) control (black) and TgF344 AD (grey) neurons measured in voltage clamp (Vm =  − 75 mV). **c**–**e** Graphs show that the cumulative frequency of peak amplitude of acetylcholine current was not different at 8 months (**c**), was significantly higher for TgF344 AD responses than controls at 12 months (*P* < 0.01) (**d**), and was not different at 18 months (**e**). Insets show the data in scattergram. **f** Representative responses to exogenous bath-applied acetylcholine from F344 WT control (black) and TgF344 AD (grey) neurons measured in current clamp. **g**, **h** Graphs show that the cumulative frequency of time to peak firing of acetylcholine response was not different at 8 months (**g**), was significantly faster for TgF344 AD responses than controls at 12 months (*P* < 0.01) (**h**), and was not different at 18 months (**i**). Insets show the data in scattergram. Rats aged in three cohorts: 8 months (nTg: 8.5 ± 0.2 months, *n* = 12 animals, 4–5 brain slices per animal, TgF344: 8.4 ± 0.2, *n* = 9 animals, 4–5 brain slices per animal), 12 months (nTg: 12.4 ± 0.2 months, *n* = 13, 4–5 brain slices per animal, TgF344 12.5 ± 0.2 months, *n* = 10 animals, 4–5 brain slices per animal), and 18 months (nTg: 17.7 ± 0.3 months, *n* = 14 animals, 4 –5 brain slices per animal, TgF344: 17.6 ± 0.2 months, *n* = 12 animals, 4–5 brain slices per animal)

To evaluate whether cholinergic upregulation in response to AD pathology is recapitulated across species, we consider the TgF344-AD rat model [57]. We compared TgF344-AD and WT littermate controls at three stages of disease: early-AD (8 months), mid-AD (12 months), and later-AD (18 months) [58–61]. Strikingly, we again found an upregulation in cholinergic currents in TgF344-AD rats compared to controls (Fig. 3a, b). This difference was specific to the mid-AD age group (KS test, *P* = 0.005, D = 0.4) (Fig. 3c–e). It was not observed in the early-AD group (KS test, *P* = 0.7, D = 0.2) or the later-AD group (KS test, *P* = 0.7, D = 0.2).

A similar pattern was observed for changes in action potential firing in response to cholinergic stimulation (Fig. 3f). The TgF344-AD rat model showed an upregulation in cholinergic firing, as measured by the reduced time taken to attain peak firing rate relative to controls at mid-AD (Fig. 3g–i, KS test, *P* = 0.003, D = 0.4). However, this difference was not observed at early-AD (KS test, *P* = 0.8, D = 0.1) or later-AD (KS test, *P* = 1, D = 0.1).

To delve deeper into the cholinergic responses, we examined the impact of sex within each age group. We detected no sex differences in ACh currents (two-way ANOVA, 8 months: *F*_1, 98_ = 0.4, *P* = 0.5; 12 months: *F*_1, 77_ = 0.04, *P* = 0.8; 18 months: *F*_1, 80_ = 0.003, *P* = 0.9) or significant interactions between sex and genotype (8 months: *F*_1, 98_ = 2.7, *P* = 0.1; 12 months: *F*_1, 77_ = 0.09, *P* = 0.8; 18 months: *F*_1, 80_ = 0.3, *P* = 0.6).

The differences in cholinergic responses observed in the mid-AD (12 month) group occurred in the absence of differences in intrinsic properties (Additional file 1: Table S2). Examination of the excitability of these neurons with input–output analysis (Additional file 1: Fig. S2a–c) showed a significant decrease in the intrinsic excitability curve of TgF344 AD neurons at 12 months (Additional file 1: Fig. S2b; nonlinear regression, comparison of fit, *F*_3,561_ = 3.8, *P* = 0.01). Interestingly, this decrease of excitability in response to current injection coincided with the emergence of increased excitability to cholinergic stimulation (Fig. 3). This finding underscores the relative specificity of increased cholinergic excitation of layer 6 neurons at mid-AD in this rat model. In the early AD (8 months) and later-AD (18 months) groups, we observed no significant differences in layer 6 neuronal properties (Additional file 1: Table S2) or intrinsic excitability (Additional file 1: Fig. S2a,c).

Together, this work replicated upregulation of cholinergic responses in layer 6 pyramidal neurons in a different AD model of a different species, and with a different method of cholinergic stimulation. This conserved property underscores the importance of understanding the receptor mechanisms.

### Upregulated responses arise from a selectively increased nicotinic signal

**Fig. 4:**
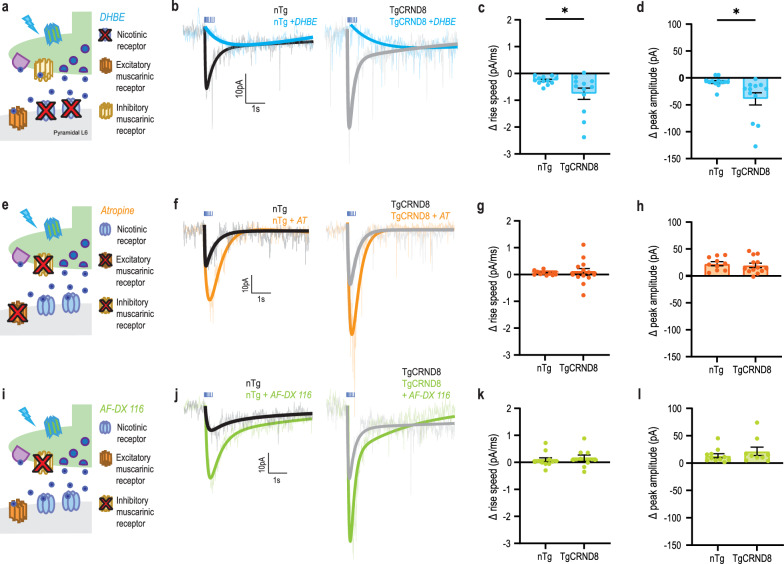
Early cholinergic upregulation in the AD model is specific to nicotinic receptor signalling. **a** Schematic depicts a cholinergic synapse in the presence of nicotinic receptor antagonist DHβE. **b** Representative opto-ACh responses from non-transgenic (nTg) control (black) and TgCRND8 AD (grey) neurons measured in voltage clamp (*V*_m_ =  − 75 mV) before and after DHβE. **c**, **d** Graphs show that DHβE caused greater declines in rise speed (*P* < 0.05) (**c**) and peak amplitude (*P* < 0.05) (**d**) of opto-ACh currents in TgCRND8 AD responses relative to controls (age range: 4.5 to 12.2 months, mean = 7.9 ± 0.7 months, *n* = 14 animals, 1–3 brain slices per animal). **e** Schematic depicts a cholinergic synapse in the presence of broad muscarinic receptor antagonist atropine. **f** Representative opto-ACh responses from non-transgenic (nTg) control (black) and TgCRND8 AD (grey) neurons measured in voltage clamp before and after atropine. Graphs show that atropine elicited changes in rising speed (**g**) and peak amplitude (**h**) that were not different between TgCRND8 AD and control responses (age range: 3.1 to 12.2 months, mean = 6.4 ± 0.7 months, *n* = 20 animals, 1–3 brain slices per animal). **i** Schematic depicts a cholinergic synapse in the presence of muscarinic M2 receptor antagonist AF-DX 116. **j** Representative opto-ACh responses from non-transgenic (nTg) control (black) and TgCRND8 AD (grey) neurons measured in voltage clamp before and after AF-DX 116. Graphs show that AF-DX 116 elicited changes in rising speed (**k**) and peak amplitude (**i**) that were not different between TgCRND8 AD and control responses (age range: 4.2 to 12.2 months, mean = 7.8 ± 0.9 months, *n* = 13 animals, 1–3 brain slices per animal)

Since we found that the AD cholinergic responses were faster and had greater amplitude than non-transgenic controls, we hypothesized that nicotinic responses underlie the genotype difference, as these ionotropic receptors impart these characteristics to the endogenous response [68, 69, 73]. To investigate pre- and post-synaptic elements in the intact prefrontal cholinergic synapses, we used the opto-TgCRND8 AD mouse model. To investigate the nicotinic receptor contribution to this upregulated opto-ACh response in AD, we pharmacologically blocked these receptors using the antagonist DHβE (3 µM) (Fig. 4a). DHβE changed the cholinergic responses in both genotypes (Fig. 4b), significantly decreasing the rise speed (paired, two-tailed *t*-test: *t* = 4.3,* P* = 0.0002, df = 23) and the peak amplitude (paired, two-tailed* t*-test: *t* = 3.5, *P* = 0.002, df = 23). However, DHβE exerted a greater effect on the AD group, yielding a significantly greater change in rising slope (Fig. 4c, unpaired two-tailed *t*-test, *t* = 2.3, *P* = 0.03, df = 22) and peak amplitude (Fig. 4d, unpaired two-tailed *t*-test, *t* = 2.6, *P* = 0.01, df = 22). These results suggest that the increased opto-ACh responses in this preclinical AD model involved a prominent upregulation in nicotinic receptor signalling.

Since G-protein coupled muscarinic receptors contribute both pre- and post-synaptically to prefrontal cholinergic synapses [68], we next evaluated whether muscarinic components of the cholinergic synapses were altered. Blocking muscarinic receptors with the broad-spectrum antagonist atropine (200 nM; Fig. 4e) increased the amplitude of cholinergic responses in both genotypes (Fig. 4f; paired two-tailed *t*-test: *t* = 7.4, *P* < 0.0001, df = 22), but did not change the rising slope (paired two-tailed *t*-test: *t* = 1.1, *P* = 0.3, df = 22). However, there was no significant genotype difference in the effect of atropine on either of these measures (rising slope: unpaired two-tailed *t*-test: *t* = 0.3, *P* = 0.8, df = 21; peak amplitude: unpaired two-tailed *t*-test: *t* = 0.5, *P* = 0.6, df = 21) (Fig. 4g, h). Consistent with our interpretation that atropine increases opto-ACh response amplitude by blocking presynaptic autoinhibition, targeted blocking of presynaptic muscarinic autoreceptors with AF-DX 116 (Fig. 4i) also increased opto-ACh responses in both AD and control (Fig. 4j). AF-DX 116 increased the opto-ACh response amplitude (paired, two-tailed *t*-test: *t* = 4.2, *P* = 0.005, df = 19) but did not change the rising slope (paired, two-tailed *t*-test: *t* = 1.7, *P* = 0.1, df = 19). There was no significant genotype difference in the effect of AF-DX 116 on either of these measures (rising slope: unpaired two-tailed *t*-test: *t* = 0.5, *P* = 0.7, df = 18; peak amplitude: unpaired two-tailed *t*-test: *t* = 1, *P* = 0.3, df = 18) (Fig. 4k, l). These findings suggest that neither pre- nor post-synaptic muscarinic receptors are substantially altered in this preclinical AD model.

### Treatment strategies: acetylcholinesterase inhibition and nicotinic potentiation amplify cholinergic upregulation

To probe the impact of treatment intervention on this upregulated cholinergic signalling, we measured the effects of current standard-of-care treatment and a novel treatment strategy on the amplitude and kinetics of the opto-ACh responses of neurons in the TgCRND8 AD mice at early-to-mid and advanced disease stages.

**Fig. 5:**
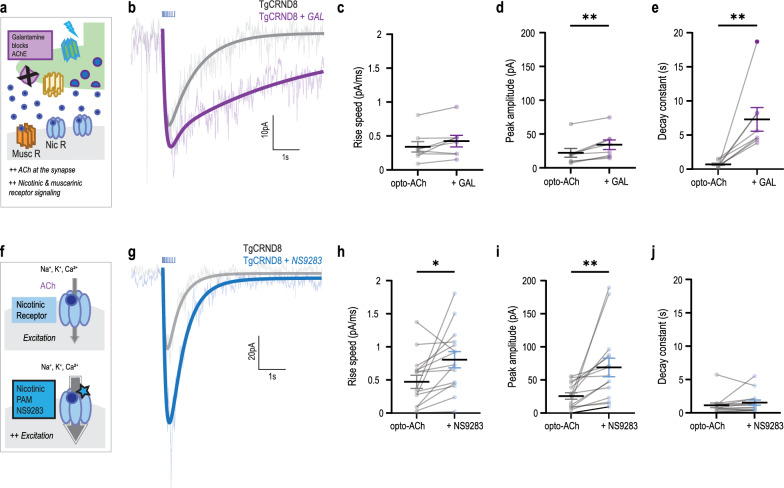
Galantamine and a novel nicotinic treatment strategy further enhance cholinergic upregulation in AD model. **a** Schematic depicts a cholinergic synapse in the presence of acetylcholinesterase inhibitor galantamine. Galantamine slowed the degradation of acetylcholine, prolonging its availability at the synapse. **b** Representative opto-ACh responses from a TgCRND8 AD neuron before (grey) and after galantamine (purple) treatment. **c**–**e** Graphs show that galantamine elicited no change in the rising speed (**c**) but significant increases in peak amplitude (*P* < 0.01) (**d**) and response duration (**e**), as measured by the decay constant (*P* < 0.01) in TgCRND8 AD responses (age range 3–6 months: mean = 5.2 ± 0.6 months, *n* = 5 animals, 1–3 brain slices per animal). **f** Schematic depicts the activation of postsynaptic nicotinic receptors by acetylcholine and the action of nicotinic positive allosteric modulator NS9283 in increasing nicotinic receptor conductance. **g** Representative opto-ACh responses from a TgCRND8 AD neuron before (grey) and after (blue) NS9283 treatment. **h**–**j** Graphs show that NS9283 elicited increases in rise speed (*P* < 0.05) (**h**) and peak amplitude (*P* < 0.001) (**i**) with no change in the response duration (**j**) (age range 3–6 months: mean = 4.7 ± 0.4 months, *n* = 6 animals, 1–3 brain slices per animal)

The pro-cognitive standard-of-care treatment galantamine inhibits acetylcholinesterase (Fig. 5a), allowing acetylcholine to remain longer at the synapse. Applying galantamine to opto-ACh AD responses (Fig. 5b) showed a trend of increasing the rising speed (Fig. 5c, paired, two-tailed *t*-test, *t* = 2.3, *P* = 0.06, df = 7) and significantly increased the peak amplitude (Fig. 5d, paired, two-tailed *t*-test, *t* = 4.4, *P* = 0.003, df = 7). However, the greatest impact of galantamine was to significantly prolong the decay constant (Fig. 5e, paired two-tailed *t*-test, *t* = 3.6, *P* = 0.008, df = 7). This effect of galantamine was consistent between TgCRND8 mice and non-transgenic controls (Additional file 1: Fig. S3a–c) and consistent in TgCRND8 mice between early-to-mid and advanced AD stages (Additional file 1: Fig. S4a–c). Galantamine modestly increases the peak response magnitude; however, it prolongs the overall kinetics of the usually brief cholinergic response.

To explore treatment interventions targeting the specific nicotinic receptor upregulation seen in this AD model, we tested the nicotinic-selective positive allosteric modulator NS9283 which enhances endogenous cholinergic signalling (Fig. 5f). We found that NS9283 greatly boosted opto-ACh AD responses (Fig. 5g), significantly increasing the rising speed (Fig. 5h; paired, two-tailed *t*-test, *t* = 2.7, *P* = 0.02, df = 14) and the peak amplitude (Fig. 5i; paired two-tailed *t*-test, *t* = 3.2, *P* = 0.007, df = 14). Strikingly, these changes occurred without changes of the decay constant (Fig. 5j; paired two-tailed *t*-test, *t* = 1.3, *P* = 0.2, df = 14) of the opto-ACh response. The action of NS9283 was consistent between TgCRND8 mice and non-transgenic controls (Additional file 1: Fig. S3d–f) and consistent in TgCRND8 mice between early-to-mid and late AD stages (Additional file 1: Fig. S4d–f). This intervention strongly amplified peak response magnitude while retaining the typical rapid and brief response kinetics.

The current standard-of-care treatment galantamine significantly increased response amplitude (71% ± 16%) and showed a trend of increase in rising speed (35% ± 16%), but at the cost of significant slowing of the cholinergic response (2032% ± 993%). In comparison, the nicotinic-specific NS9283 induced significant enhancement of the rising speed (210% ± 68%) and a significant increase in amplitude (249% ± 87%), and modestly but not consistently prolonged response decay (77% ± 32%). There results suggest that NS9283 incites a greater increase in response magnitude and better conserves endogenous response timing and kinetics than the broad cholinergic standard-of-care treatment. Therefore, it is possible to use a targeted treatment such as NS9283 to further enhance upregulation of this cognitively crucial signalling pathway.

## Discussion

In this study, we demonstrate a functional upregulation of layer 6 prefrontal cholinergic signalling in preclinical models of AD. Cholinergic upregulation occurs during early to mid-disease progression in two species models with differing pathology trajectories. Elevated functional nicotinic signalling plays a dominant role, and the apparent compensation is sensitive to further enhancement via pharmacological manipulation. The AD treatment with acetylcholinesterase inhibition boosts the cholinergic response but slows its time course. In contrast, targeted nicotinic positive allosteric modulation exerts a strong and significant potentiation with preserved kinetics. Taken together, our results suggest a potential active mechanism of cognitive compensation and demonstrate an effective approach to amplify it and potentially extend it to the late AD stage.

### Cognitive reserve and neural compensation in AD

Cognitive reserve, or the brain’s ability to sustain neurological decline while maintaining cognitive processes, is well documented clinically in aging [12, 13, 76, 77] and in AD [78, 79]. Studies have explored the neurophysiological basis for cognitive resilience in AD [15, 80] including in the prefrontal cortex [81]. In preclinical models, plasticity related to AD has been detected in cortical neuronal morphology [82–89] and in neurotransmitter signalling [90, 91], including acetylcholine neuromodulation [92]. In the TgF344 AD rat model used here, for example, parvalbumin interneuron neuroplasticity is observed at mid-pathology, with coinciding cognitive resilience in executive function and cognitive flexibility [14]. Interestingly, here we found cholinergic upregulation in layer 6 prefrontal cortex at the same mid-disease timepoint.

Prefrontal cholinergic signalling is crucial for many cognitive functions maintained by cognitive reserve, including attention, cue detection, and working memory [18–20, 22, 23]. The nicotinic excitation of layer 6 pyramidal neurons is thought to play a key role in attention and cue detection [21, 24]. The importance of prefrontal nicotinic signalling in successful attention [93–96] becomes evident under demanding conditions, where locally stimulating nicotinic receptors improves performance [97] and deficient prefrontal nicotinic signalling worsens performance [21, 24, 98]. These cognitive processes are timescale-dependent [99–101], where configurations of the fast ionotropic nicotinic receptor that include the α5 subunit lead to faster responses to endogenous acetylcholine [69] and contribute to better attentional performance [21]. The nicotinic positive allosteric modulator NS9283, which potentiates cholinergic responses while maintaining fast endogenous kinetics, improves performance on attention tasks in wild-type rodents [102, 103].

Clinically, in individuals pre-diagnosed with elevated amyloid-β (Aβ) and none/mild baseline cognitive deficits, cholinergic inhibition with scopolamine unmasks otherwise undetectable cognitive deficits that correlate with Aβ load [104]. This supports that in pre-diagnosis or early AD, cholinergic signalling may be important in compensating for pathological decline and maintaining cognitive functions. This preclinical and clinical behavioral literature, coupled with the opto-physiological data in the present study, underscores the need for future work to examine the behavioral impact of nicotinic-targeted therapeutics such as NS9283 in models of AD and, particularly, its interactions with age and stage of disease.

### Cholinergic signalling and synapses in AD

Previous molecular investigations into cholinergic neurons and synapses in AD emphasize a trajectory of cholinergic decline, with decreases in both pre- and post-synaptic markers resulting in eventual heterogenous loss of cholinergic neurons in advanced AD [32, 33, 105–115]. However, much of this characterization was performed later in disease progression [116, 117]. Molecular and functional investigations that have been carried out in early to mid-disease mostly consist of swiftly-progressing genetic models of AD that preclude the use of littermate controls [38–44]. To detect and systematically investigate mechanisms of compensatory plasticity, it is essential to use a well-charted model with appropriately matched non-transgenic controls. Furthermore, functional characterisation is vital for understanding cholinergic synaptic transmission.

Here, we found upregulation of prefrontal cholinergic signalling in layer 6 that emerges after disease onset, at early to mid-disease in two different preclinical models of AD. Both the TgCRND8/ChATChR2 AD mice and the TgF344 AD rats harbor human *APP* mutations [48, 57], resulting in amyloid-driven AD pathologies. Aβ interacts with nicotinic receptors [118–125], the consequences of which depend on cell type and nicotinic receptor configuration. Nicotinic receptors are regulated by many cellular elements, including lynx family proteins [73, 126] and protein kinase C by way of muscarinic receptor agonism [68], providing many potential avenues for signalling to be harnessed for compensation. Our use of models with non-transgenic littermate controls revealed an upregulation of nicotinic signalling in early to mid-AD that did not continue to increase in later-AD. Strengths of our electrophysiological and opto-physiological approaches include functional insight into cholinergic signalling upregulation and identification of this upregulation as specific to nicotinic receptors.

A caveat for our work is its limitation to layer 6 pyramidal neurons. While this neuronal population has prominent expression of nicotinic receptors and their regulatory lynx prototoxins [73] and plays a key role in attention [21, 24, 98], processes of cognitive reserve likely also require the involvement of neurons in other prefrontal layers. It will be important for future work to specifically target the impact of endogenous cholinergic responses on additional neurophysiological processes beyond those involved in attention.

### Translational and clinical impact

Deficits in attention and executive function have consequences for other cognitive domains: if you cannot attend, you cannot encode [127]. It is therefore important to identify mechanisms through which we can improve attention and extend the maintenance of this cognitive domain as much as possible throughout AD progression. Nicotinic signalling has long been a target for enhancing cognition, including in AD [128, 129]. Direct stimulation of nicotinic receptors is pro-cognitive in AD [27], potentially protective against Aβ [130, 131] but complicated by desensitization [132–134]. Nicotinic positive allosteric modulators, such as NS9283 used here, present less risk of desensitization [135], and are pro-cognitive preclinically [102, 103]. Our results suggest that nicotinic positive allosteric modulation further enhances nicotinic signalling upregulation in AD.

Attention and cue-detection are mediated by cholinergic, and specifically nicotinic stimulation of the prefrontal cortex [19, 20, 93–96, 136] and depend on fast ionotropic receptor kinetics [21, 69]. Acetylcholinesterase inhibitor, a current and long-used AD treatment, enhances all cholinergic signaling: fast, ionotropic nicotinic signal and slower, longer-lasting metabotropic muscarinic signal shown above (Fig. 5) and previously [68, 69]. While acetylcholinesterase inhibitor imparts some increase in response amplitude, it profoundly slows response decay. Treatments that maintain the endogenous timing of responses, such as NS9283 that increases the pro-attentional rise kinetics, largely amplifies response magnitude, and does not alter decay, might better enhance time-sensitive cognitive functions.

Understanding neural compensation and neurological changes at different disease stages is important for understanding the implications of clinical interventions. Different levels of effectiveness of novel AD treatments may result from timing of intervention and the level of neurological changes already set in motion by AD. Adaptations that are pro-cognitive in the presence of AD may have altered outcomes if pathology is resolved. Therefore, it is important to take neurological changes into account when determining treatment strategies.

## Conclusion

Overall, we uncover evidence for cholinergic compensation in two preclinical models of AD. In both models, functional upregulation in prefrontal cholinergic signalling occurs in early to mid-AD. Mechanistic investigation reveals increased nicotinic signalling, which is amenable to further upregulation, both by acetylcholinesterase inhibitor and by nicotinic positive allosteric modulation. While the scope of benefit across cognitive domains requires investigation, harnessing compensatory nicotinic upregulation may be an important treatment strategy to further enhance and preserve cognition in AD.

## Supplementary Information

**Additional file 1**: **Table S1** Neuronal intrinsic properties of layer 6 pyramidal neurons by genotype in mice. **Table S2** Neuronal intrinsic properties of layer 6 pyramidal neurons by genotype in rats. **Figure S1** There are no significant differences in intrinsic excitability between nTg and TgCRND8 layer 6 pyramidal neurons in mouse.  **Figure S2** Decrease in intrinsic excitability between F344 and TgF344 layer 6 pyramidal neurons in mid disease.  **Figure S3** Cholinergic treatments are consistently effective in prefrontal layer 6 pyramidal neurons of TgCRND8 AD and nTg controls. **Figure S4** Cholinergic treatments are consistently effective at early to mid-AD and late-AD in layer 6 pyramidal neurons of prefrontal cortex

## Data Availability

The datasets generated during and/or analysed during the current study are not publicly available due to agency over intellectual property but are available from the corresponding author on reasonable request.

## Abbreviations

ACh: Acetylcholine
AD: Alzheimer’s disease
AT: Atropine
ChAT: Choline acetyl transferase
ChR2: Channelrhodopsin
DHβE: Dihydro-β-erythroidine
Gal: Galantamine
Opto-ACh: Optogenetic release of acetylcholine
