# Supplementary material for: Enhanced prefrontal nicotinic signaling as evidence of active compensation in Alzheimer’s disease models
Source: Transl Neurodegener. 2024 Dec 3;13:58. doi: 10.1186/s40035-024-00452-7 (PMC11613856; doi:10.1186/s40035-024-00452-7)
Supplement: Supplementary file 1 — Additional file 1: Table S1 Neuronal intrinsic properties of layer 6 pyramidal neurons by genotype in mice. Table S2 Neuronal intrinsic properties of layer 6 pyramidal neurons by genotype in rats. Figure S1 There are no significant differences in intrinsic excitability between nTg and TgCRND8 layer 6 pyramidal neurons in mouse. Figure S2 Decrease in intrinsic excitability between F344 and TgF344 layer 6 pyramidal neurons in mid disease. Figure S3 Cholinergic treatments are consistently effective in prefrontal layer 6 pyramidal neurons of TgCRND8 AD and nTg controls. Figure S4 Cholinergic treatments are consistently effective at early to mid-AD and late-AD in layer 6 pyramidal neurons of prefrontal cortex [file 40035_2024_452_MOESM1_ESM.pdf]

Enhanced prefrontal nicotinic signaling as evidence of active compensation in Alzheimer's disease models

Saige K Power[1], Sridevi Venkatesan[1], Sarah Qu[1], JoAnne McLaurin[2,3], Evelyn K Lambe[1,4,5]

Supplemental Materials

- Supplemental Tables S1 & S2
- Supplemental Figures S1, S2, S3, & S4

Supplemental Table S1

	Capacitance	Input resistance	Resting membrane potential	Threshold	Spike amplitude
nTg<br>3-6 months	85 ± 2 pF	137 ± 5 MΩ	-85 ± 1 mV	-50 ± 1 mV	63 ± 3 mV
TgCRND8<br>3-6 months	87 ± 3 pF	127 ± 6 MΩ	-84 ± 1 mV	-50 ± 1 mV	69 ± 2 mV
Statistics	$t_{(112)}$ = 0.5<br>P = 0.6	$t_{(112)}$ = 1.1<br>P = 0.3	$t_{(112)}$ = 0.6<br>P = 0.6	$t_{(112)}$ = 0.5<br>P = 0.6	$t_{(112)}$ = 1.8<br>P = 0.08
Summary	*NS*	*NS*	*NS*	*NS*	*NS*
nTg<br>7-10 months	87 ± 1 pF	143 ± 6 MΩ	-80 ± 3 mV	-50 ± 1 mV	67 ± 2 mV
TgCRND8<br>7-10 months	89 ± 2 pF	160 ± 9 MΩ	-82 ± 2 mV	-48 ± 1 mV	65 ± 3 mV
Statistics	$t_{(99)}$ = 0.7<br>P = 0.5	$t_{(99)}$ = 1.5<br>P = 0.1	$t_{(99)}$ = 0.6<br>P = 0.6	$t_{(99)}$ = 1.2<br>P = 0.2	$t_{(99)}$ = 0.7<br>P = 0.5
Summary	*NS*	*NS*	*NS*	*NS*	*NS*

Neuronal intrinsic properties of layer 6 pyramidal neurons by genotype in mouse. Table shows mean ± SEM for each intrinsic property in neurons from non-transgenic (nTg) and TgCRND8 AD model mice at early/mid AD (3 to 6 months) and later AD (7-10 months), as well as the results of the unpaired *t*-tests comparing genotypes in each age group.

Supplemental Table S2

	Capacitance	Input resistance	Resting membrane potential	Threshold	Spike amplitude
F344 nTg 8 months	104 ± 4 pF	96 ± 4 MΩ	-91 ± 1 mV	-52 ± 1	72 ± 1 mV
TgF344 8 months	96 ± 4 pF	101 ± 5 MΩ	-91 ± 1 mV	-52 ± 1	69 ± 1 mV
Statistics	$t_{(107)}$ = 1.5 P = 0.1	$t_{(107)}$ = 0.4 P = 0.7	$t_{(107)}$ = 0.5 P = 0.6	$t_{(107)}$ = 0.05 P = 0.9	$t_{(107)}$ = 1.7 P = 0.9
Summary	*NS*	*NS*	*NS*	*NS*	*NS*
F344 nTg 12 months	101 ± 4 pF	104 ± 7 MΩ	-89 ± 1 mV	-51 ± 1	72 ± 2 mV
TgF344 12 months	95 ± 3.5 pF	87 ± 4 MΩ	-90 ± 1 mV	-50 ± 1	67 ± 2 mV
Statistics	$t_{(85)}$ = 0.5 P = 0.6	$t_{(85)}$ = 1.9 P = 0.06	$t_{(85)}$ = 0.2 P = 0.9	$t_{(85)}$ = 1.3 P = 0.2	$t_{(85)}$ = 1.8 P = 0.08
Summary	*NS*	*NS*	*NS*	*NS*	*NS*
F344 nTg 18 months	105 ± 4 pF	112 ± 6 MΩ	-88 ± 1 mV	-49 ± 1	67 ± 2 mV
TgF344 18 months	95 ± 4 pF	127 ± 8 MΩ	-88 ± 1 mV	-50 ± 1	69 ± 1 mV
Statistics	$t_{(110)}$ = 1.8 P = 0.07	$t_{(110)}$ = 1.5 P = 0.1	$t_{(110)}$ = 0.4 P = 0.7	$t_{(110)}$ = 1.4 P = 0.1	$t_{(110)}$ = 1.4 P = 0.2
Summary	*NS*	*NS*	*NS*	*NS*	*NS*

Neuronal intrinsic properties of layer 6 pyramidal neurons by genotype in rat. Table shows mean ± SEM for each intrinsic property in neurons from F344 non-transgenic (nTg) and TgF344 AD model rats at each age point, as well as the results of the unpaired *t*-tests comparing genotypes at each age point.

Supplemental Figure S1

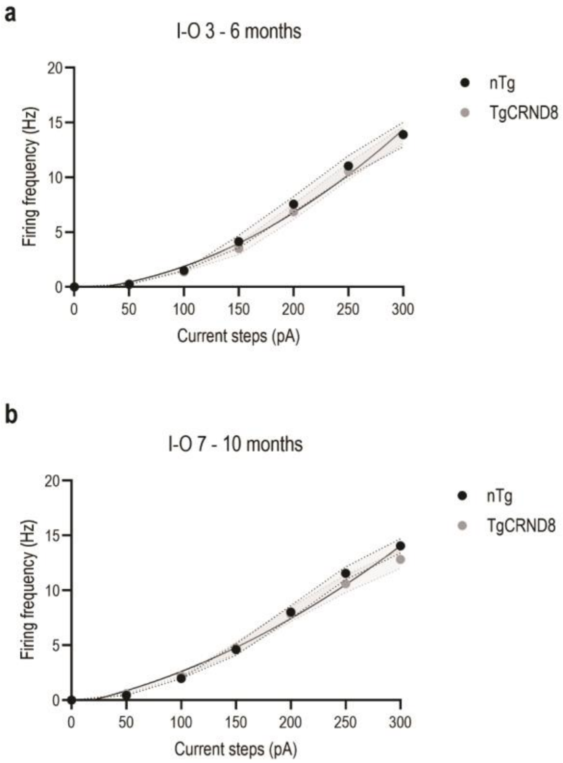

There are no significant differences in intrinsic excitability between nTg and TgCRND8 layer 6 pyramidal neurons in mouse. Input-output graphs show the firing frequency of pyramidal layer 6 prefrontal cortex neurons in response to increasing steps of injected current in **a** neurons from 3 to 6 month old TgCRND8 and nTg animals and **b** neurons from 7 to 10 month old TgCRND8 and nTg animals. There is no significant difference between intrinsic excitability of neurons of the younger (nonlinear regression, comparison of fit, $F_{3,750} = 0.4$, $P = 0.7$) nor older age group ($F_{3,788} = 1.6$, $P = 0.1$).

Supplemental Figure S2

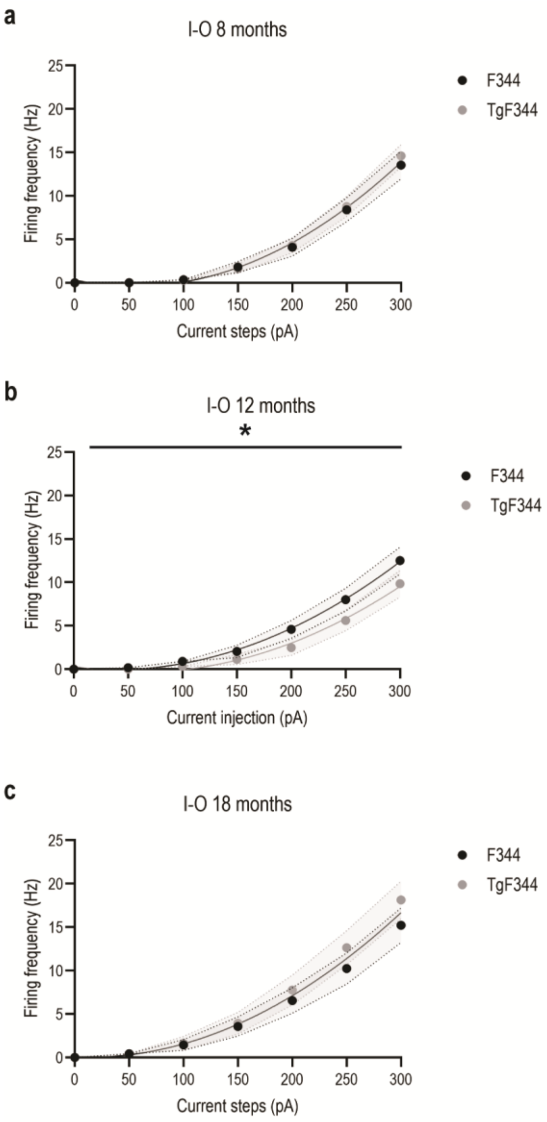

Decrease in intrinsic excitability between F344 and TgF344 layer 6 pyramidal neurons in mid disease. Input-output graphs show the firing frequency of pyramidal layer 6 prefrontal cortex neurons in response to increasing steps of injected current in neurons from TgF344 and F344 control animals at **a** 8 month old, **b** 12 month old, and **c** 18 month old. At 12 months, there is a significant decrease in intrinsic excitability of TgF344 AD neurons (nonlinear regression, comparison of fit, $F_{3,561} = 3.8$, $P = 0.01$). This coincides with the emergence of increased cholinergic excitability (see **Fig. 3**). There are no significant differences in intrinsic excitability of pyramidal L6 neurons at 8 months ($F_{3,603} = 0.3$ $P = 0.8$) nor 18 months, ($F_{3,449} = 1.5$, $P = 0.2$).

Supplemental Figure S3

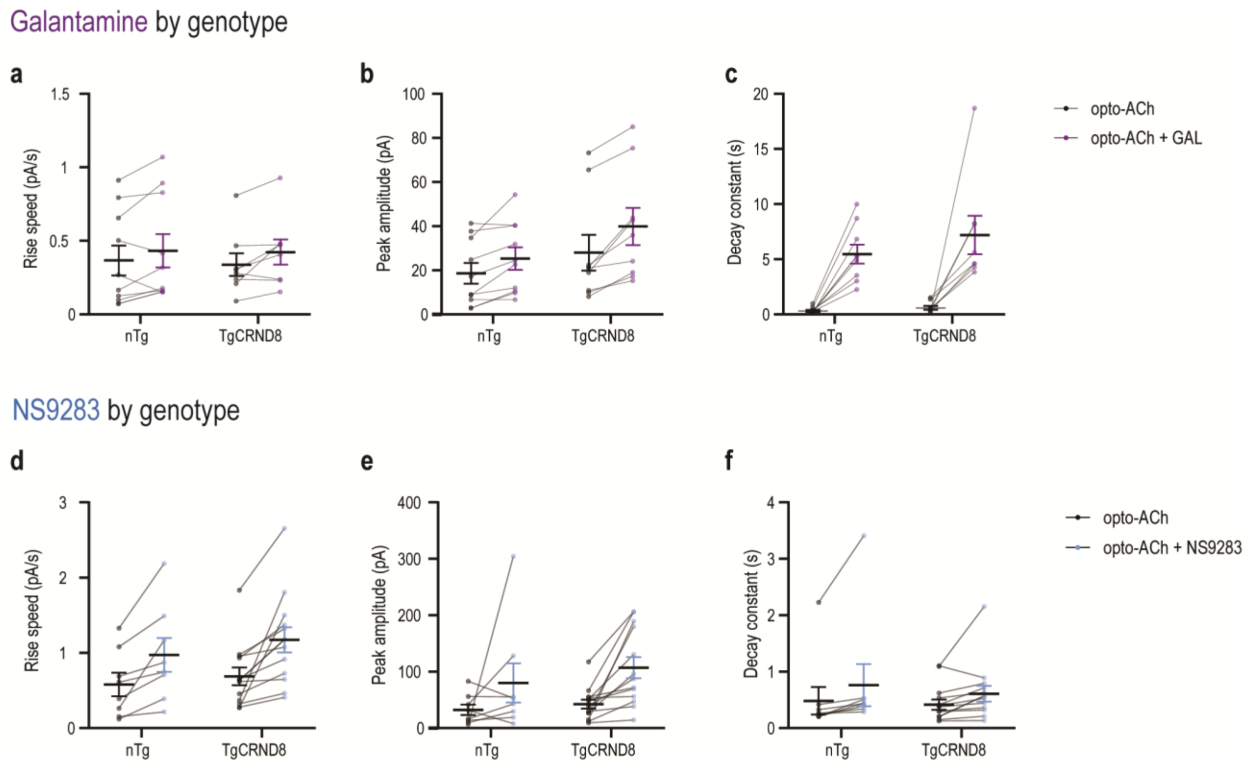

Cholinergic treatments are consistently effective in prefrontal layer 6 pyramidal neurons of TgCRND8 AD and nTg controls. Graphs show the effect of galantamine (purple) and NS9283 (blue) on opto-ACh responses in non-transgenic controls (nTg) as compared to TgCRND8 mice. Galantamine elicits no significant effect on **a** rise speed or **b** peak amplitude in either genotype but does elicit a significant drug effect on **c** decay constant ($P < 0.0001$, drug effect, two-way ANOVA) (nTg: 4 ± 0.4 months, n = 6 animals, 1–2 brain slices per animal; TgCRND8: 5.2 ± 0.6 months, n = 5 animals, 1–2 brain slices per animal). NS9283 elicits a significant drug effect on **d** rise speed ($P = 0.01$, drug effect, two-way ANOVA) and **e** peak amplitude ($P = 0.006$, drug effect, two-way ANOVA) for both genotypes with no significant effect on **f** decay (nTg: 4.1 ± 0.7 months, n = 5 animals, 1–2 brain slices per animal; TgCRND8: 4.7 ± 0.4 months, n = 6 animals, 1–2 brain slices per animal). There are no interactions between genotype and drug treatment.

Supplemental Figure S4

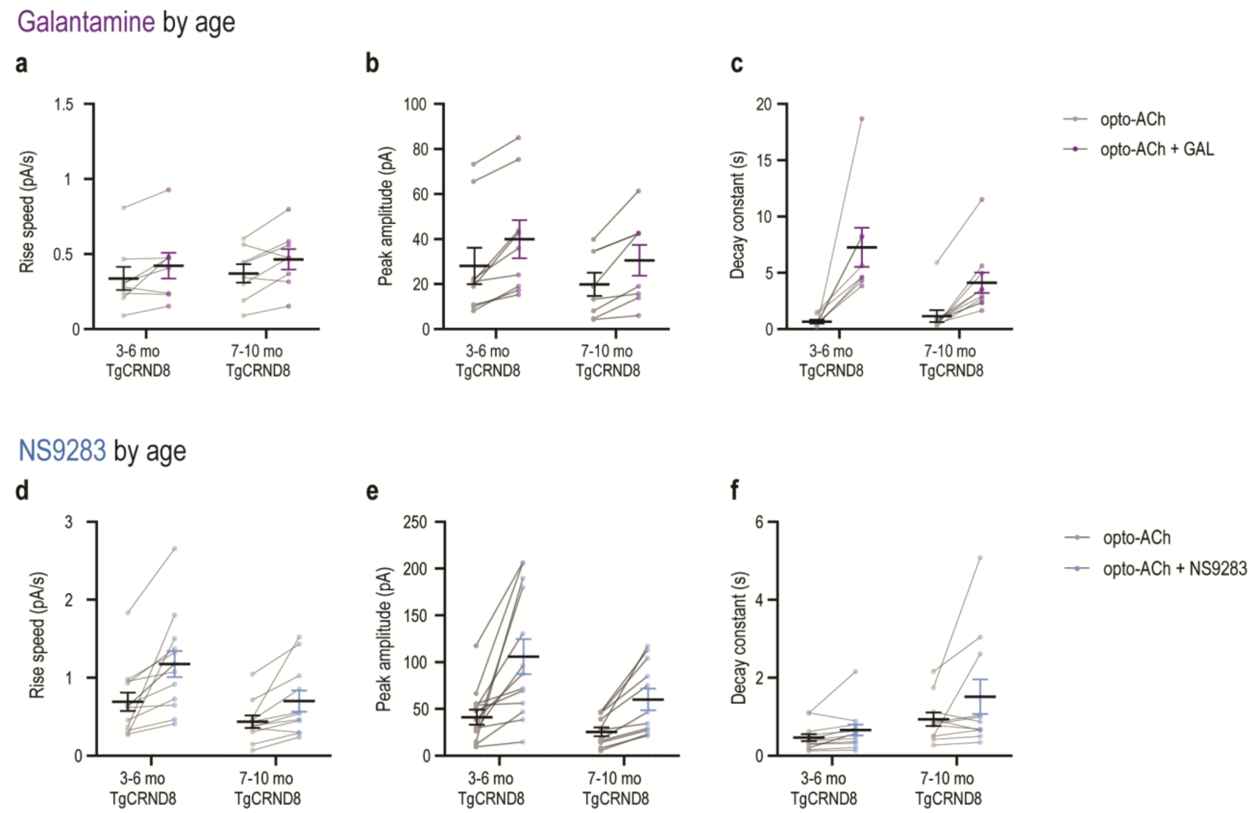

Cholinergic treatments are consistently effective at early to mid-AD and late-AD in layer 6 pyramidal neurons of prefrontal cortex. Graphs show the effect of galantamine (purple) and NS9283 (blue) on opto-ACh responses in early to mid AD TgCRND8 mice (3-6 months) as compared to late AD TgCRND8 mice (7-12 months). Galantamine elicits no significant drug effect on **a** rise speed or **b** peak amplitude for either age point but elicits a significant drug effect on **c** decay ($P < 0.0001$, drug effect, two-way ANOVA) (3-6 months: 5.2 ± 0.6 months, n = 5 animals, 1–2 brain slices per animal; 7-12 months: 8.5 ± 0.3, n = 4 animals, 1–2 brain slices per animal). NS9283 elicits a significant effect on **d** rise speed ($P = 0.0007$, drug effect, $P = 0.0009$, age effect, two-way ANOVA) and **e** peak amplitude ($P = 0.0003$, drug effect, $P = 0.02$, age effect, two-way ANOVA) for both age points with no significant effect on **f** decay (3-6 months: 4.7 ± 0.4 months, n = 6 animals, 1–2 brain slices per animal; 7-12 months: 8.3 ± 0.7 months, n = 5 animals, 1–2 brain slices per animal). There are no interactions between age and drug treatment.
